# Association of HLA-DR1, HLA-DR13, and HLA-DR16 Polymorphisms with Systemic Lupus Erythematosus: A Meta-Analysis

**DOI:** 10.1155/2022/8140982

**Published:** 2022-04-16

**Authors:** Tingrui Wang, Hong Wang, Lijuan Qiu, Lingling Wu, Huayun Ling, Yu Xue, Ying Zhou, Huijuan Chen, Dong-Qing Ye, Bin Wang

**Affiliations:** ^1^Department of Epidemiology and Biostatistics, School of Public Health, Anhui Medical University, Hefei, Anhui, China; ^2^Inflammation and Immune Mediated Diseases Laboratory of Anhui Province, Hefei, Anhui, China

## Abstract

**Objectives:**

The principal purpose of this meta-analysis was to assess the association between HLA-DRB1 (HLA-DR1, HLA-DR13, and HLA-DR16) polymorphisms and SLE susceptibility.

**Methods:**

We searched published case-control studies on the association between HLA-DRB1 polymorphisms and SLE susceptibility from PubMed and Web of Science databases. The pooled ORs with 95% CIs were utilized to estimate the strength of association of HLA-DR1, HLA-DR13, and HLA-DR16 polymorphisms and SLE susceptibility by fixed effect models. We also performed sensitivity analysis, trial sequential analysis, Begg's test, and Egg's test in this meta-analysis.

**Results:**

A total of 18 studies were included in this meta-analysis. Overall analysis showed that HLA-DR1 and HLA-DR13 polymorphisms were associated with a decreased risk of SLE (OR = 0.76, 95% CI: 0.65-0.90, *P* < 0.01; OR = 0.58, 95% CI: 0.50-0.68, *P* < 0.01), and HLA-DR16 polymorphism was associated with an increased risk of SLE (OR = 1.70, 95% CI: 1.24-2.33, *P* < 0.01). In subgroup analysis of ethnicity, the results were as follows: HLA-DR1 polymorphism in Caucasians (OR = 0.76, 95% CI: 0.58-0.98,*P* = 0.04) and North Americans (OR = 0.64, 95% CI: 0.42-0.96,*P* = 0.03); HLA-DR13 polymorphism in Caucasians (OR = 0.62, 95% CI: 0.47-0.82,*P* < 0.01) and East Asians (OR = 0.44, 95% CI: 0.34-0.57,*P* < 0.01); and HLA-DR16 polymorphism in East Asians (OR = 2.62, 95% CI: 1.71-4.03,*P* < 0.01).

**Conclusions:**

This meta-analysis showed that HLA-DR1 and HLA-DR13 are protective factors for SLE, and HLA-DR16 is a risk factor. Due to the limitations of this meta-analysis, the association between HLA-DRB1 polymorphisms and SLE susceptibility needs to be further researched before definitive conclusions are proved.

## 1. Introduction

Systemic lupus erythematosus (SLE) is a chronic autoimmune disease with recurrence and remission, characterized by the production of autoantibodies and the deposition of immune complexes, which can lead to irreversible damage of multiple organ systems [1, 2]. SLE mainly occurs in women with a female to male ratio of approximately 10 : 1 [3], which has an incidence of 0.3 to 31.5 cases per 100,000 individuals and a prevalence of 3.2 to 517.5 cases per 100,000 individuals in the world population [4]. The pathogenesis of SLE is multifactorial, involving the interaction of genetic and environmental factors [5]. The human leukocyte antigen (HLA) system, which is also known as the major histocompatibility complex (MHC), plays a key role in the antigen presentation of intracellular and extracellular peptides and the regulation of innate and adaptive immune responses [6, 7]. The HLA region, which contains the six classic HLA genes, is the most polymorphic region in the human genome, and the HLA genes are also the most relevant gene among all SLE susceptibility genes [8]. The HLA genes involved in the immune response are divided into two classes, including class I HLA-A, -B, and-C and class II HLA-DRB1, -DQB1, and -DPB1, which are different in structure and function [9, 10], although genetic studies have found a strong association between HLA-DRB1 gene polymorphism and autoimmune diseases [11]. Due to the high polymorphism of HLA genes and the linkage imbalance between HLA loci, it is difficult to define the specific association between HLA genes and SLE [12]. Previous studies have shown that HLA-DRB1 polymorphisms have a significant association with SLE susceptibility [13, 14]. However, the relationship between HLA-DRB1 polymorphisms and SLE susceptibility is complex. Further research is needed to clarify the exact mechanism of this relationship.

To more deeply evaluate the relationship between HLA-DR1, HLA-DR13, and HLA-DR16 gene polymorphisms and SLE susceptibility and systematically analyze their role in the pathogenesis of SLE, we conducted a meta-analysis with the purpose of drawing statistically valid conclusions through the analysis of a large number of samples and providing possible directions for further research.

## 2. Literature Search

We conducted a comprehensive search for studies assessing the relationship between HLA-DRB1 polymorphisms and SLE susceptibility until September 1, 2021. All publications related to the research theme are obtained through systematic retrieval of PubMed and Web of Science databases. The following keywords and Mesh terms were combined to improve the sensitivity and specificity of the search strategy: “systemic lupus erythematosus”, “SLE”, “human lymphocyte antigen”, “HLA”, “HLA-DRB”, “HLA-DRB1”, “polymorphism”, “SNP”, “single nucleotide polymorphism”, “variation”, and “mutation”. Meanwhile, we manually searched the references of the retrieved literatures and relevant reviews to collect relevant literatures to the greatest extent. There were no restrictions on region and publication time.

### 2.1. Study Selection

All studies included in this meta-analysis need conform to the following inclusion criteria: (1) a case-control study to appraise the association between HLA-DRB1 polymorphisms and SLE susceptibility; (2) with sufficient available data to estimate an odds ratio (OR) with 95% confidence interval (95% CI); (3) for multiple studies conducted in the same population or subpopulation, the latest or most complete publications shall be selected; and (4) all cases met the diagnostic criteria for SLE revised by the American College of Rheumatology (ACR) in 1982 or 1997.

The studies were carefully screened by the following exclusion criteria: (1) subject irrelevant study, review, or meta-analysis; (2) letter, comment, editorial, meeting, or abstract; (3) animal study; and (4) study was a genetic linkage analysis of family members.

### 2.2. Data Extraction

The two researchers independently extracted detailed data from the identified literature and cross-checked to ensure that the information collected was accurate. Any disputes in data extraction or evaluation were settled through group discussion or arbitrated by the study leader. The particular information extracted from each study included the following aspects: first author, publication year, country or region studied, ethnicity of the studied population, matching criteria for controls, number of cases and controls, mean age, genotype or allele frequency, diagnostic standard, and genotyping method.

### 2.3. Statistical Analysis

The intensity of the association between the three polymorphisms and SLE risk was assessed by calculating the combined ORs with 95% CIs, and the heterogeneity among independent research results was examined by Cochran's *Q* statistics and inconsistency index (*I*^2^) statistics. The percentage of inter-study variation in the overall variation is quantified by *I*^2^ statistic, the greater the *I*^2^ statistic and the greater the heterogeneity. The low, medium, and high degrees of heterogeneity are represented by the *I*^2^ statistics 25%, 50%, and 75%, respectively. If there is no heterogeneity or low (*P* > 0.10, *I*^2^ < 50%) between the studies, the fixed effect model is used for the combined analysis of the data; otherwise, the random effect model is applied. Begg's test and Egg's test were used to evaluate the potential publication bias. When *P* was less than 0.05, it indicated that results of the research are biased. To evaluate the ethnicity-specific effect and improve the robustness of study, subgroup analysis was performed by ethnicity categorized as Caucasian, North American, African, South Asian, and East Asian. The robustness and dependability of the research results were also appraised by sensitivity analysis. All statistical analyses were carried out using the Stata SE 12 software (Stata Corporation, College Station, TX, United States). The *P* values of the two-sided test were less than 0.05, which is considered statistically significant.

### 2.4. Trial Sequential Analysis

Trial sequential analysis (TSA) is a method to calculate *Z*-curve, TSA boundary value, traditional boundary value, and required information size (RIS) by using a relative risk reduction (RRR) of 20%, a power of 80%, and a type I error of 5%. TSA is mainly used to evaluate the risk of type I error in meta-analysis and whether there is sufficient sample size to draw the current conclusion. The analysis was performed using the TSA software version 0.9.5.10 Beta (Copenhagen Trial Unit, Copenhagen, Denmark).

## 3. Results

### 3.1. Literature Search and Characteristics of Eligible Studies

**Figure 1:**
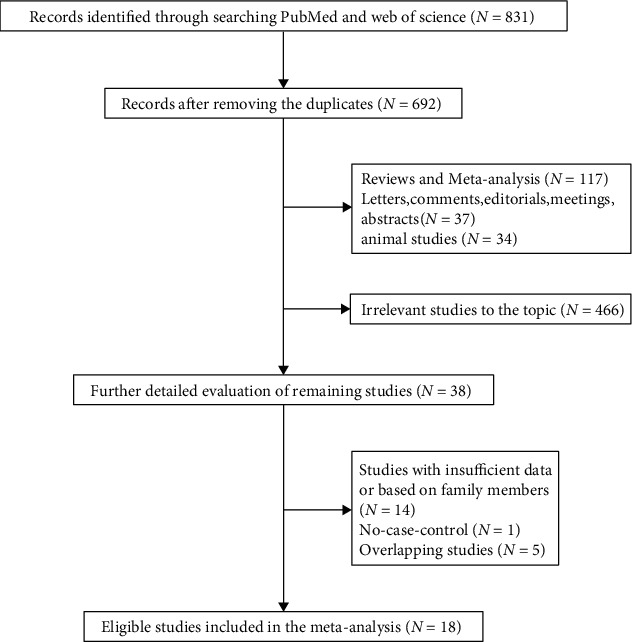
Flow chart for the literature search and screening in this meta-analysis.

According to the retrieval strategy, we collected a total of 831 literatures that may be related to the research from the electronic database, including 510 records from PubMed and 321 records from Web of Science. 139 duplicates were deleted through review, and the remaining records were retained. Then, we carefully examined the titles and abstracts of the rest of the literature; 654 records were excluded based on the exclusion criteria. Finally, we critically evaluated the remaining 38 articles through careful review of the full text. 20 studies failed to meet the requirements, and the remaining 18 studies [15–32] that met the inclusion criteria were finally included in this meta-analysis. The specific reasons for the exclusion and detailed screening flow chart are shown in Figure 1. The particular characteristics of these 18 qualified studies in this meta-analysis are summarized in Table 1. Newcastle-Ottawa Scale (NOS) was used to assess the quality of the included studies.  Table 2 shows that all included studies had a score of 6 or more, so they were considered high-quality studies with low risk of bias.

### 3.2. Association of HLA-DR1, HLA-DR13, and HLA-DR16 Polymorphisms with SLE Susceptibility

**Figure 2:**
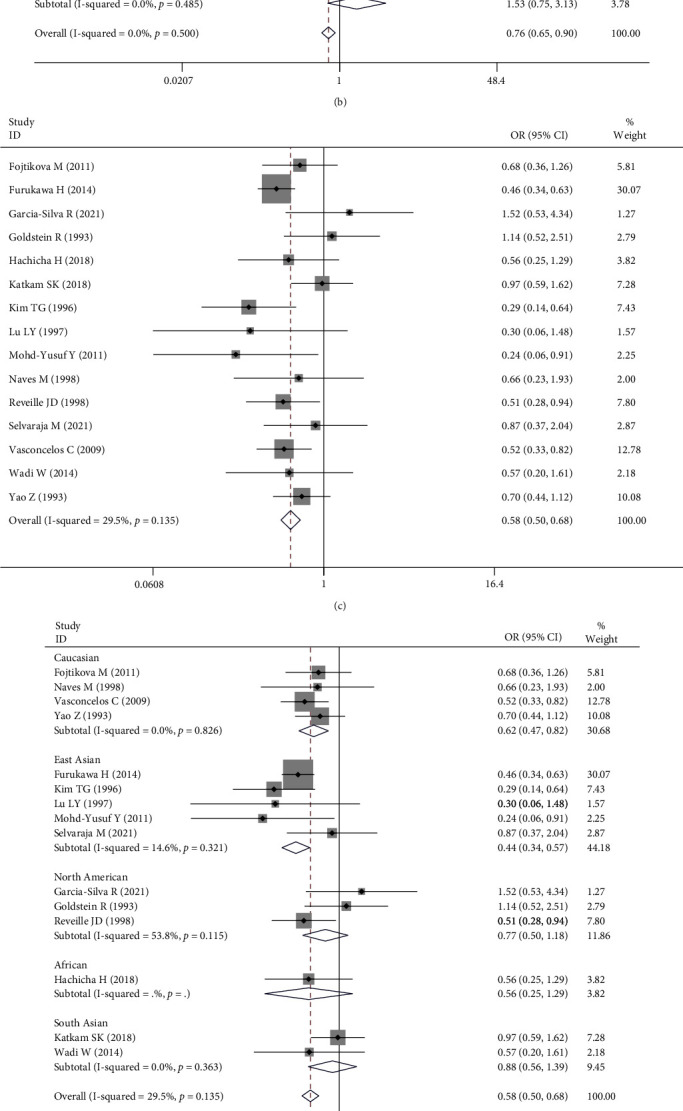
Forest plot of SLE risk associated with HLA-DRB1 polymorphisms in this meta-analysis. (a) Overall analysis of SLE risk associated with HLA-DR1 polymorphism. (b) Subgroup analysis (ethnicity) of SLE risk associated with HLA-DR1 polymorphism. (c) Overall analysis of SLE risk associated with HLA-DR13 polymorphism. (d) Subgroup analysis (ethnicity) of SLE risk associated with HLA-DR13 polymorphism. (e) Overall analysis of SLE risk associated with HLA-DR16 polymorphism. (f) Subgroup analysis (ethnicity) of SLE risk associated with HLA-DR16 polymorphism.

Table 3 shows that HLA-DR1 and HLA-DR13 polymorphisms were associated with a reduced risk of SLE (OR = 0.76, 95% CI: 0.65-0.90, *P* < 0.01; OR = 0.58, 95% CI: 0.50-0.68, *P* < 0.01), and HLA-DR16 polymorphism was associated with an increased risk of SLE (OR = 1.70, 95% CI: 1.24-2.33, *P* < 0.01) (Figures 2(a), 2(c), and 2(e)).

In subgroup analysis of ethnicity, the results were as follows: HLA-DR1 was associated with SLE in Caucasians (OR = 0.76, 95% CI: 0.58-0.98, *P* = 0.04) and North Americans (OR = 0.64, 95% CI: 0.42-0.96, *P* = 0.03) (Figure 2(b)); HLA-DR13 was associated with SLE in Caucasians (OR = 0.62, 95% CI: 0.47-0.82, *P* < 0.01) and East Asians (OR = 0.44, 95% CI: 0.34-0.57, *P* < 0.01) (Figure 2(d)); HLA-DR16 was associated with SLE in East Asians (OR = 2.62, 95% CI: 1.71-4.03, *P* < 0.01) (Figure 2(f)).

### 3.3. Sensitivity Analysis

**Figure 3:**
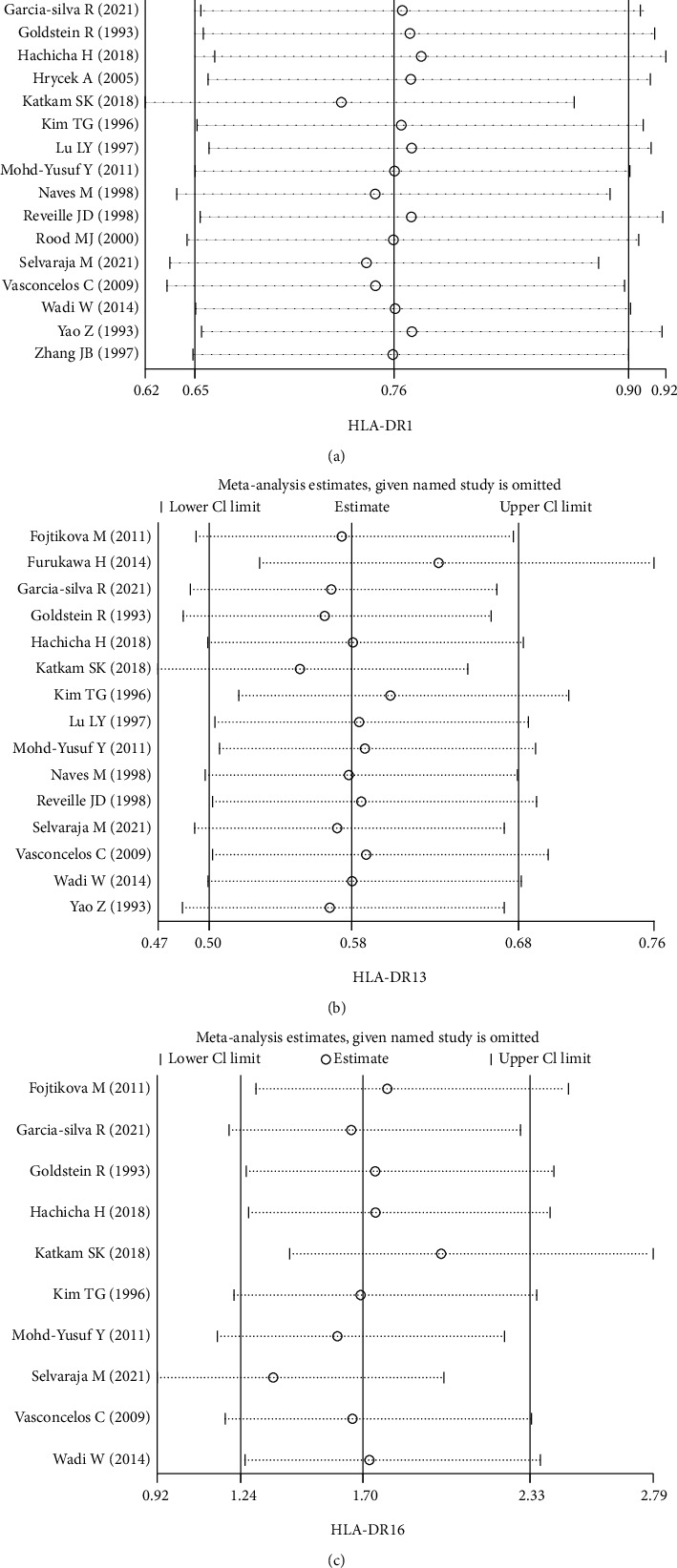
Sensitivity analysis of SLE risk associated with HLA-DR1 (a), HLA-DR13 (b), and HLA-DR16 (c) polymorphisms.

We conducted a sensitivity analysis to detect the impact of each study on the overall meta-analysis and evaluate the stability of the overall meta-analysis results of the three gene polymorphisms. When we ignored each included study in turn, the corresponding statistics (ORs with 95% CIs) had not been substantially changed, which indicated that the results of the meta-analysis were comparatively stable and dependable (Figure 3).

### 3.4. Publication Bias

The publication bias of this meta-analysis was detected by Begg's test and Egg's test. The results were as follows: HLA-DR1 (*P* value of Egg's test was 0.589, and *P* value of Begg's test was 0.405), HLA-DR13 (*P* value of Egg's test was 0.641, and *P* value of Begg's test was 0.843), and HLA-DR16 (*P* value of Egg's test was 0.050, and *P* value of Begg's test was 0.210). All *P* values were not less than 0.05 indicated that there was no striking evidence of publication bias in this meta-analysis (Table 3).

### 3.5. Trial Sequential Analysis

**Figure 4:**
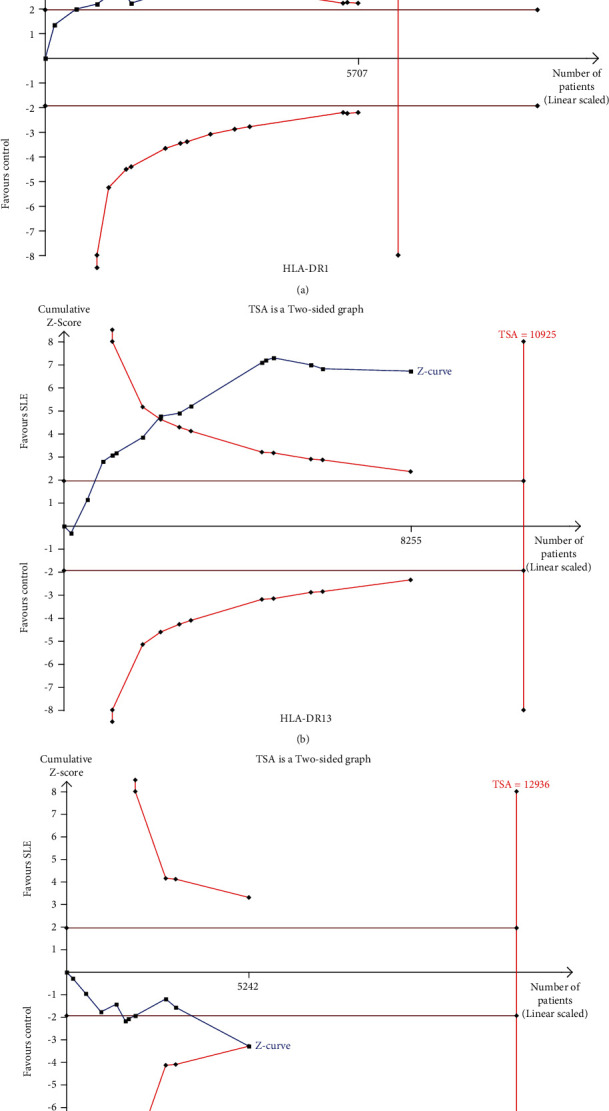
Trial sequential analysis of SLE risk associated with HLA-DR1 (a), HLA-DR13 (b), and HLA-DR16 (c) polymorphisms.

The results of TSA showed that the cumulative *Z*-curve of HLA-DR1, HLA-DR13, and HLA-DR16 has crossed TSA boundary value and traditional boundary value, and the cumulative *Z*-curve of HLA-DR1 also has exceeded RIS, which indicated that the research results have reached a reliable conclusion (Figure 4).

## 4. Discussion

As an autoimmune disease involving multiple systems, SLE often causes irreversible harm to multiple organ systems and affects the life span and quality of life of patients [33, 34]. Epidemiological studies show that there are obvious regional differences in incidence, prevalence, immunology, and clinical changes of SLE, which may have a bearing on different genetic and environmental factors [35]. The HLA system, which located on the short arm of chromosome 6 (6p21.3), encodes at least two hundred genes [36]. The most polymorphic gene cluster in the human genome is the HLA system, which plays an indispensable role in resisting pathogens and affecting the development of autoimmune diseases [37]. All SLE GWAS in different populations, through the in-depth study of the association between HLA variation and SLE susceptibility, have proved that HLA regions are the most remarkable and strongest predictors of genetic risk [38]. As the main candidate gene of SLE susceptibility, HLA class II gene is strictly related to the pathogenesis of SLE, which contains HLA-DRB1, HLA-DQB1, and HLA-DPB1 [9]. Up to now, a considerable number of literatures have discussed the potential relationship between HLA-DRB1 polymorphisms and SLE. Previous studies have shown that the allele frequencies of HLA-DR1 and HLA-DR13 in Korean SLE patients are significantly lower [39, 40], and HLA-DRB1∗13 : 02 in Japanese SLE patients are also significantly reduced [41]. A study on Mexican SLE patients found that the frequency of HLA-DRB1∗16 : 01 haplotype increased [42]. We conducted this meta-analysis to explore the potential association between HLA-DRB1 polymorphisms and SLE susceptibility as much as possible.

In this meta-analysis, we evaluated published evidence for the association between HLA-DR1, HLA-DR13, and HLA-R16 polymorphisms and susceptibility to SLE. The results showed HLA-DR1 and HLA-DR13 were protective factors for SLE, which can reduce the risk of disease. On the contrary, HLA-DR16 was associated with an increased risk of SLE as a risk factor. Due to the genetic background varying among populations, the effects of gene-gene and gene-environment interactions and the strong linkage disequilibrium of biologically related variants would lead to genetic heterogeneity among populations [43]. Consequently, to evaluate the race-specific effect, we performed a subgroup analysis by ethnicity to determine whether target gene polymorphisms in a specific population are associated with SLE susceptibility. We found that HLA-DR1 was significantly associated with SLE susceptibility in Caucasians and North Americans, and HLA-DR13 was in East Asians and Caucasians. Due to insufficient publications that met the requirements, we only found a significant association between HLA-DR16 and the risk of SLE in East Asians. The reason for the difference in disease-specific association may be that the specific MHC class II alleles (especially HLA-DR and HLA-DQ alleles) change the targeting of specific autoantigens in T cell-dependent antibody responses [44]. Our subgroup analysis showed that ethnicity had an important impact on SLE susceptibility.

On the one hand, risk MHC class II molecules increase the risk of disease by allowing pathogenic autoreactive T cells to escape from central tolerance, and protective MHC class II molecules confer disease resistance by promoting negative selection and the development of autoreactive regulatory T-cells (Tregs) [45, 46]. These processes are controlled by the binding affinity of T-cell receptors (TCRs) to peptide-MHC (pMHC) [46]. Antigenic peptides are presented by MHC class II molecules to T cell receptors (TCRs) on homologous T cells. The increased affinity between pMHC presented by HLA-DR1 and HLA-DR13 and TCRs leads to enhance negative selection and agonist selection of Tregs, thereby inhibiting autoimmune response. On the contrary, the low affinity between pMHC presented by HLA-DR16 and TCRs results in defective negative selection and Treg developmental, which eventually leads to autoimmune diseases. This may be the underlying mechanism of the influence of HLA-DR1, HLA-DR13, and HLA-DR16 on SLE susceptibility. On the other hand, HLA-DRB1 molecules carry three risk residues (positions 11, 13, and 26) located in the peptide-binding groove for disease susceptibility of specific autoantibodies against autoantigens associated with SLE, which affect the interaction between amino acids of autoantigens and amino acids conforming to peptide-binding groove in HLA molecule and antigen presentation for T cell activation [47, 48]. Amino acid haplotype decreased SLE risk in HLA-DR1 (11Leu-13Phe-26Leu) and HLA-DR13 (11Ser-13Ser-26Phe) and increased SLE risk in HLA-DR16 (11Pro-13Arg-16Phe) [48], which showed that the residues in peptide binding groove of HLA-DRB1 molecule may have an impact on SLE susceptibility. In addition, HLA-DRB1∗13 was associated with IgG and IgM antibodies against *β*2GP-1 [49], and HLA-DRB1∗16 was positively associated with antinuclear and anti-Sm antibodies [47]. Those indicated that the effect of HLA-DRB1 on autoantibody formation was also closely related to the pathogenesis of SLE to a certain extent. Considering the strong linkage disequilibrium of HLA region and the complexity of pathogenesis, the definite role of HLA-DRB1 in the pathogenesis of SLE needs to be further explored in the future.

The component studies in this meta-analysis were case-control studies with enough publicly available data to estimate ORs and the corresponding 95% CIs. By subgroup analysis, the statistical power and the accuracy of effect estimates are improved. However, only one study from Africans was included, and the association between HLA-DRB1 polymorphisms and susceptibility to SLE in Africans requires further research in the future. Meanwhile, there are several limitations in this meta-analysis. Firstly, according to the search strategy, we only searched English literature in the two databases, so the potential publication bias was inevitable. Secondly, although age, gender, and environment variables have important effects on the pathogenesis of SLE, we did not conduct subgroup analysis due to the lack of sufficient data. Thirdly, our ethnic-specific meta-analysis was mainly conducted in Caucasian, Asian, and North American. Therefore, our results are more applicable to these populations, and the conclusion needs to be further enhanced and demonstrated in more in-depth research. Finally, considering these limitations, we should be cautious about the results of the association between HLA-DRB1 polymorphisms and SLE susceptibility.

In conclusion, the gene frequencies of HLA-DR1, HLA-DR13, and HLA-DR16 in SLE patients and healthy individuals were remarkably different, which suggested that HLA-DR1 and HLA-DR13 are protective factors for SLE, and HLA-DR16 is a risk factor. Due to the limitations of this meta-analysis, the association between HLA-DRB1 polymorphisms and SLE susceptibility needs to be further researched before definitive conclusions are proved.

## Figures and Tables

**Table 1:** Characteristics of included studies in this meta-analysis.

Study	Year	Country/area	Ethnicity	Matching criteria	Sample size		Mean age		Diagnostic criteria	Detection methods
					SLE	Control	SLE	Control		
Fojtíková et al. [15]	2011	Czech Republic	Caucasian	Age	123	99	43.4	39.3	ACR	PCR-SSP
Furukawa et al. [16]	2014	Japan	East Asian	Age	848	849	45.2	36.0	ACR	PCR-SSOP
Garcia-Silva et al. [17]	2021	Mexico	North American	Ethnicity	40	99	30	NA	ACR	PCR-SSP
Goldstein and Sengar [18]	1993	Canada	North American	NA	91	89	NA	NA	ACR	RFLP
Hachicha et al. [19]	2018	Tunisia	African	Age	75	123	32	32	ACR	PCR-SSP
Hrycek et al. [20]	2005	Poland	Caucasian	Age	24	36	41	36	ACR	PCR-SSP
Katkam et al. [21]	2018	India	South Asian	Ethnicity + age	212	227	28.04	30.00	ACR	PCR-SSP
Kim et al. [22]	1996	Korea	East Asian	Ethnicity	97	281	NA	NA	ACR	PCR-SSP
Lu et al. [23]	1997	Taiwan	East Asian	Ethnicity	105	115	29.2	NA	ACR	PCR-SSP
Mohd-Yusuf et al. [24]	2011	Malaysia	East Asian	NA	160	107	NA	NA	ACR	PCR-SSP
Naves et al. [25]	1998	Spain	Caucasian	Age + sex	46	47	NA	NA	ACR	PCR-SSOP
Reveille et al. [26]	1998	USA	North American	NA	225	393	35.6	NA	ACR	PCR-SSOP
Rood et al. [27]	2000	Netherlands	Caucasian	Ethnicity	99	177	NA	NA	ACR	PCR-SSOP
Selvaraja et al. [28]	2021	Malaysia	East Asian	NA	100	951	30.6	NA	ACR	PCR-SSOP
Vasconcelos et al. [29]	2009	Portugal	Caucasian	Ethnicity	213	223	NA	NA	ACR	PCR-SSP
Wadi et al. [30]	2014	Saudi Arabia	South Asian	NA	51	30	NA	NA	ACR	PCR-SSP
Yao et al. [31]	1993	Germany	Caucasian	NA	178	207	NA	NA	ACR	PCR-SSOP
Zhang et al. [32]	1997	China	East Asian	NA	51	106	NA	NA	ACR	PCR-SSP

ACR: American College of Rheumatology Criteria; PCR: polymerase chain reaction; SSP: sequence-specific primers; SSOP: sequence-specific oligonucleotide probe; RFLP: restriction fragment length polymorphism; NA: not available.

**Table 2:** Quality of included studies was assessed according to the Newcastle-Ottawa scale.

Study	Adequate definition of cases	Representativeness of cases	Selection of control subjects	Definition of control subjects	Control for important factor	Exposure assessment	Same method of ascertainment for all subjects	Nonresponse rate	Total
Fojtíková et al. [15]	1	0	1	1	1	1	1	1	7
Furukawa et al. [16]	1	1	0	1	1	1	1	1	7
Garcia-Silva et al. [17]	1	1	1	1	1	1	1	1	8
Goldstein and Sengar [18]	1	1	1	1	0	1	1	0	6
Hachicha et al. [19]	1	0	1	1	1	1	1	1	7
Hrycek et al. [20]	1	1	1	1	1	1	1	1	8
Katkam et al. [21]	1	0	1	1	2	1	1	1	8
Kim et al. [22]	1	0	1	1	1	1	1	1	7
Lu et al. [23]	1	0	1	1	1	1	1	1	7
Mohd-Yusuf et al. [24]	1	0	1	1	0	1	1	1	6
Naves et al. [25]	1	1	1	1	2	1	1	0	8
Reveille et al. [26]	1	1	1	1	0	1	1	0	6
Rood et al. [27]	1	1	0	1	1	1	1	1	7
Selvaraja et al. [28]	1	1	1	1	0	1	1	1	7
Vasconcelos et al. [29]	1	1	1	1	1	1	1	0	7
Wadi et al. [30]	1	0	1	1	0	1	1	1	6
Yao et al. [31]	1	1	1	1	0	1	1	0	6
Zhang et al. [32]	1	0	1	1	0	1	1	1	6

**Table 3:** Results of the association between HLA-DRB1 (HLA-DR1, HLA-DR13, and HLA-DR16) polymorphisms and SLE susceptibility in this meta-analysis.

Subgroup	No. of studies	Sample size		Test of association		Test of heterogeneity		*P*_Egg's test_	*P*_Begg's test_
		SLE	Control	OR (95% CI)	*P*	*I* ^2^ (%)	*P*		
HLA-DR1									
Overall	18	2738	4159	0.76 (0.65, 0.90)	<0.01	0	0.500	0.589	0.405
Caucasian	6	683	789	0.76 (0.58, 0.98)	0.04	0	0.705		
East Asian	6	1361	2409	0.79 (0.60, 1.05)	0.11	26.8	0.234		
North American	3	356	581	0.64 (0.42, 0.96)	0.03	0	0.913		
African	1	75	123	0.45 (0.18, 1.10)	0.08	NA	NA		
South Asian	2	263	257	1.53 (0.75, 3.13)	0.25	0	0.485		
HLA-DR13									
Overall	15	2564	3840	0.58 (0.50, 0.68)	<0.01	29.5	0.135	0.641	0.843
Caucasian	4	560	576	0.62 (0.47, 0.82)	<0.01	0	0.826		
East Asian	5	1310	2303	0.44 (0.34, 0.57)	<0.01	14.6	0.321		
North American	3	356	581	0.77 (0.50, 1.18)	0.23	53.8	0.115		
African	1	75	123	0.56 (0.25, 1.29)	0.17	NA	NA		
South Asian	2	263	257	0.88 (0.56, 1.39)	0.59	0	0.363		
HLA-DR16									
Overall	10	1162	2229	1.70 (1.24, 2.33)	<0.01	37.6	0.108	0.050	0.210
Caucasian	2	336	322	1.46 (0.71, 3.00)	0.30	43.4	0.184		
North American	2	131	188	1.69 (0.70, 4.09)	0.24	0	0.388		
African	1	75	123	0.54 (0.06, 5.29)	0.60	NA	NA		
South Asian	2	263	257	0.53 (0.21, 1.36)	0.19	0	0.951		
East Asian	3	357	1339	2.62 (1.71, 4.03)	<0.01	0	0.720		

NA: not available.

## Data Availability

The data supporting this meta-analysis are from previously reported studies and datasets, which have been cited. The processed data are available from the corresponding author upon request.
